# Prognostic impact of TROP2 in adenocarcinoma of the esophageal junction and stomach

**DOI:** 10.1007/s12094-025-04203-6

**Published:** 2026-01-14

**Authors:** Anselm Pittrof, Alexander Arnold, Severin Daum, Jonas Staudacher, Erika Berg, Michael Hummel, David Horst, Beate Rau, Ulrike Stein, Lisa Marie Eich, Christoph Treese

**Affiliations:** 1 Department of Hematology, Oncology and Cancer Immunology, Charité - Universitätsmedizin Berlin, Campus Benjamin Franklin, Berlin, Germany; 2 Institute of Pathology, Charité - Universitätsmedizin Berlin, Berlin, Germany; 3 Department of Gastroenterology, Infectious Diseases and Rheumatology, Charité - Universitätsmedizin Berlin, Campus Benjamin Franklin, Berlin, Germany; 4 Department of Surgery, Charité - Universitätsmedizin Berlin, Campus Virchow-Klinikum and Campus Mitte, Berlin, Germany; 5 Experimental and Clinical Research Center, Charité – Universitätsmedizin Berlin and Max-Delbrück-Center for Molecular Medicine in the Helmholtz Association, Berlin, Germany

**Keywords:** TROP2, Trophoblast cell surface antigen 2, Gastric cancer, Esophageal cancer, AEG/S, Sacituzumab govitecan

## Abstract

**Introduction:**

Adenocarcinoma of the esophageal junction and stomach (AEG/S) remains one of the deadliest cancers worldwide. New treatment options are urgently needed. A new target could be trophoblast cell surface protein 2 (TROP2), which is expressed in a variety of solid tumors and can be targeted, e.g., by sacituzumab govitecan, which has shown promising results in triple-negative breast cancer. This study investigates the expression of TROP2 in patients with AEG/S and correlates its expression with clinical and histopathological endpoints.

**Methods:**

TROP2 expression was assessed in a cohort of 250 patients who underwent primary surgery for AEG/S. Immunohistochemistry was performed on tissue microarrays constructed from primary tumors and lymph node metastases to quantify TROP2 expression intensity. Clinical variables, including overall survival and patient demographics, as well as tumor-specific characteristics such as stage and grade, were correlated with TROP2 expression to evaluate its potential prognostic relevance in AEG/S.

**Results:**

TROP2 was expressed in 86% of primary tumors and 81.3% of lymph node metastases. The intensity of TROP2 expression (low vs. medium vs. high) was correlated negatively with overall survival (*p* < 0.05, 70.9 months vs. 54.2 months vs. 39.5 months), lymphatic invasion (*p* = 0.05, *V* = 0.138), and higher grading (*p* = 0.037, *V* = 0.143). The intensity of TROP2 expression in lymph node metastases and primary tumors correlated significantly (*p* < 0.001, *ρ* = 0.444). There was a non-significant increase in positive lymphonodal status (*p* = 0.093, *V* = 0.138) in patients with higher TROP2 expression.

**Conclusion:**

In Caucasian AEG/S patients, TROP2 is expressed in the majority of cases. TROP2 expression intensity itself has an impact on survival, which could be explained by a more aggressive phenotype, which leads to lymphatic invasion and lymph node metastasis.

## Introduction

Adenocarcinomas of the esophagogastric junction and stomach (AEG/S) remain a major global health burden, accounting for more than one million deaths annually, with the highest incidence observed in Asian populations [1]. Despite the introduction of targeted therapies—including anti-HER2 [2], anti-VEGFR2 [3], anti-Claudin 18.2 [4, 5], and anti–PD-L1 agents [6]—prognosis remains poor, with approximately half of the patients surviving beyond 1 year after diagnosis [7].

Trophoblast cell surface antigen 2 (TROP2) is a transmembrane glycoprotein involved in calcium signaling and essential physiological processes such as fetal lung development [8]. In cancer, TROP2 is frequently overexpressed and implicated in tumorigenesis, proliferation, lymphatic invasion, and metastatic spread. Elevated TROP2 expression has been reported across multiple epithelial malignancies, including breast, thyroid, and prostate cancer, where it is associated with more aggressive clinical behavior and worse outcomes [9].

Sacituzumab govitecan, an antibody–drug conjugate targeting TROP2, delivers the cytotoxic payload SN-38 directly to TROP2-expressing tumor cells, exerting potent antitumor activity and enabling a bystander effect on neighboring cells [10, 11]. Clinical trials, including ASCENT [12] and IMMU-132-01 [13], have demonstrated substantial efficacy across several epithelial cancers, resulting in regulatory approval by the U.S. Food and Drug Administration and the European Medicines Agency for metastatic triple-negative breast cancer and urothelial carcinoma.

Although the IMMU-132-01 trial included only a limited number of gastric (*n* = 5) and esophageal (*n* = 19) cancers, emerging evidence indicates that most gastric and esophageal adenocarcinomas express TROP2, with moderate-to-high expression detected in more than half of cases [14]. A large Chinese cohort comprising 600 gastric cancer patients similarly demonstrated high TROP2 expression, which correlated with poorer survival, advanced TNM stage, larger tumor size, and increased rates of lymph node and distant metastasis [15]. Preclinical data further suggest a link between TROP2 expression and therapeutic response to sacituzumab govitecan, underscoring its potential relevance as both a prognostic biomarker and therapeutic target in AEG/S [16].

To better define the prognostic significance of TROP2 and assess its potential therapeutic implications in Caucasian patients with AEG/S, robust survival data are needed. In this study, a cohort of 250 patients with AEG/S was anlyzed, assessing TROP2 expression by immunohistochemistry and correlating its expression with detailed clinical and histopathological parameters.

## Materials and methods

### Patients

Clinical data from 250 patients with AEG/S of all tumor stages, primarily treated by surgery between 1992 and 2004 at the Charité—Universitätsmedizin Berlin, were collected retrospectively. The mean follow-up was 121.7 months (95% CI 113.9–129.5). The data including patient characteristics and follow-up information were retrieved from the patient management software (SAP) and the regional population-based cancer registry (“Gemeinsames Krebsregister”) and are summarized in Table 1. This study was approved by the Institutional Review Board of the Charité (EA4/115/10).

**Table 1:** Patient characteristics of the analyzed patient cohort and distribution of TROP2-positive and -negative primary tumors

		Trop2 low	%	Trop2 mid	%	Trop2 high	%	*p* value* (2x)	N	Cramer’s V
Gender male		91	57	40	25	28	18	0.195	159	0.114
Gender female		54	59	15	16	22	24		91	
Alter < 65		82	60	27	20	28	20	0.627	137	0.061
Age ≥ 65		63	56	28	25	22	19		113	
Localization AEG		29	67	7	16	7	16	0.381	43	0.088
Localization GC		116	56	48	23	43	21		207	
Death by tumor	Yes	71	50	36	25	35	25	**0.013**	142	**0.189**
	No	70	69	18	18	14	14		102	
	Unknown								4	
Grading	1	0	0	1	100	0	0	**0.037**	1	**0.143**
	2	35	57	19	31	7	11		61	
	3	109	59	34	18	43	23		186	
	Unknown								2	
Lauren	Intestinal	51	57	25	28	13	15	0.196	89	0.110
	Diffuse	74	58	22	17	32	25		128	
	Mixed	19	61	7	23	5	16		31	
	Unknown								2	
T	1	24	73	7	21	2	6	0.135	33	0.154
	2	61	59	18	17	25	24		104	
	3	25	39	23	36	16	25		64	
	4	17	57	6	20	7	23		30	
	Unknown								17	
N	0	41	60	19	28	8	12	0.364	68	0.114
	1	44	56	19	24	16	20		79	
	2	31	58	9	17	13	25		53	
	3	29	58	8	16	13	26		50	
Nodal status	Negative	41	60	19	28	8	12	0.093	68	0.138
	Positive	104	57	36	20	42	23		182	
M	M0	119	62	38	20	35	18	0.459	192	0.079
	M1	34	52	17	26	15	23		66	
	Unknown								2	
L	L0	55	68	16	20	10	12	**0.050**	81	**0.161**
	L1	81	54	30	20	38	26		149	
	Unknown								20	
V	V0	93	62	28	19	28	19	0.331	149	0.099
	V1	41	53	17	22	20	26		78	
	Unknown								23	
CPS	< 5	113	59	44	23	33	17	0.553	190	0.070
	≥ 5	28	56	10	20	12	24		50	
	Unknown								10	
Claudin 18.2	Negative	96	59	31	19	35	22	0.282	162	0.101
	Positive	48	55	24	28	15	17		87	
	Unknown								44	
Her2neu	Negative	111	59	45	24	32	17	0.325	188	0.105
	Positive	7	44	4	25	5	31		16	
	Unknown								46	
MSI	dMMR	12	46	8	31	6	23	0.394	26	0.087
	pMMR	131	59	46	21	44	20		221	
	Unknown								3	

Positive tumors were divided by H-score in three groups (low expression 0–100, medium expression 101–200, high expression 201–300). Significant differences between the groups (*p*-value ≤ 0.05) are written in bold

“AEG” = adenocarcinoma of the esophagogastric junction; “GC” = gastric cancer; “T” = tumor, “N” = node, “M” = metastasis, “L” = lymphatic invasion, “V” = vascular invasion from the TNM staging system; “CPS” = combined positivity score of PD-L1 expression on tumor and immune cells; “MSI” = microsatellite instability, “pMMR” = proficient mismatch repair, “dMMR” = deficient mismatch repair

Significance calculated by *X*^2^ or Fisher’s exact test when appropriate

### Tissue samples

Out of formalin-fixed, paraffin-embedded (FFPE) tumor samples from 414 patients (primary tumors *n* = 314, synchronous lymph node metastasis *n* = 151), tissue micro-arrays (TMA) were engineered and analyzed histomorphologically as described before [17]. Two hundred fifty primary tumor samples and one hundred seven lymph node metastases were evaluable after processing.

TMA blocks were sectioned at 2-μm thickness for immunohistochemical staining. Antigen retrieval was performed using either “CC1 mild buffer” (Ventana Medical Systems) with heat treatment at 100 °C for 30 min or enzymatic digestion with protease 1 for 8 min. Following antigen retrieval, tissue sections underwent primary antibody incubation for 60 min at room temperature using anti-TROP2 (clone EPR20043; Abcam, diluted 1:1000). Signal detection was achieved through the avidin–biotin complex methodology with 3,3'-diaminobenzidine as chromogen. All immunohistochemical procedures were carried out using the BenchMark XT automated immunostainer (Ventana Medical Systems).

Expression was evaluated by an immunoreactivity score (H-score) [18, 19] which incorporates the percentage of tumor cells showing membranous staining and the intensity of that staining: The percentage of positive tumor cells is estimated and assigned a value from 0 to 100. The intensity of staining is scored as 0 (none), 1 (weak), 2 (moderate), or 3 (strong):

$$\begin{aligned} H{\text{ - score}} & \, = \, \left[ {\left( {\% \,{\text{of cells with weak intensity}}} \right) \times 1} \right] \\ & \quad + \left[ {\left( {\% \,{\text{with moderate intensity}}} \right) \times 2} \right] \\ & \quad + \left[ {\left( {\% \,{\text{with strong intensity}}} \right) \, \times \, 3} \right] \\ \end{aligned}$$

**Fig. 1:**
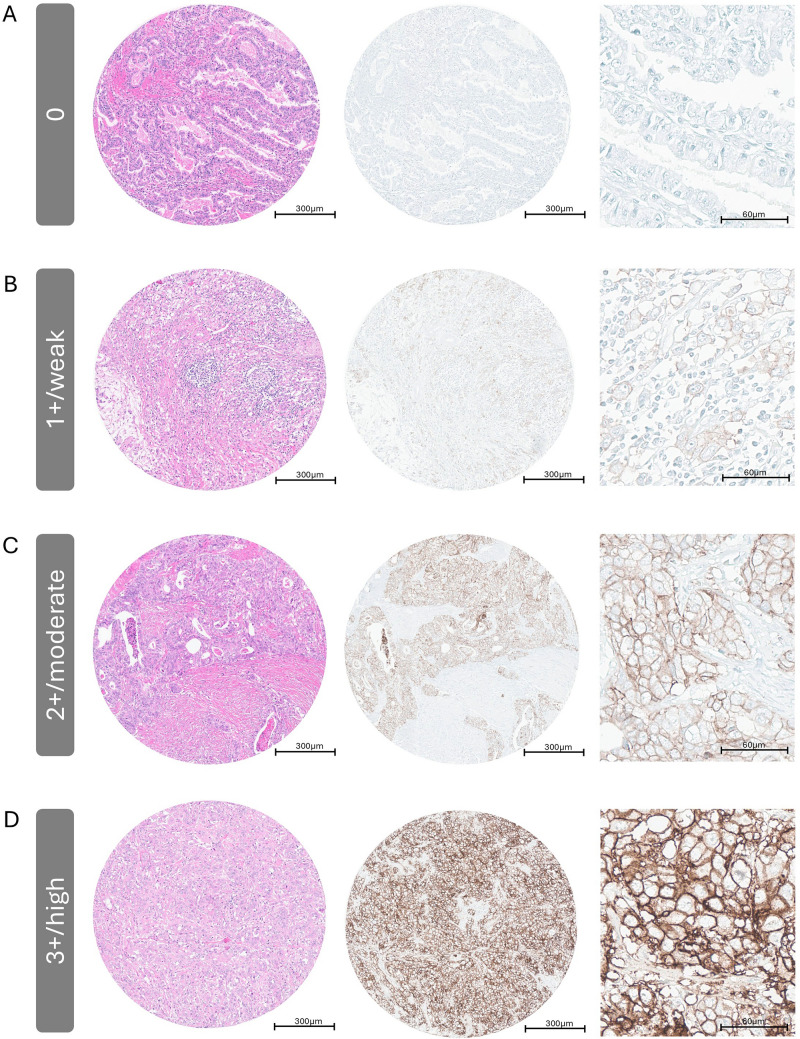
TROP2 expression in patients with adenocarcinomas of the esophagogastric junction and stomach. H&E (left) and low (middle) and high-magnification (right) histopathological images with **A** no TROP2 expression, **B** TROP2 expression of 1 + (weak), **C** TROP2 expression of 2 + (moderate) and **D** TROP2 expression of 3 + (high)

The results ranged from 0 to 300. Values over 0 were evaluated as positive TROP2 expression. The intensity of TROP2 expression was divided into three groups: low expression (0–100), moderate expression (101–200) and high expression (201–300) (For different expression levels, see Fig. 1).

The expression profiles of Claudin 18.2, HER2, and PD-L1, as well as the MSI status were previously analyzed by Arnold et al. [5].

### Statistics

Statistical analysis was performed using IBM SPSS Version 29. Overall survival was defined as time from diagnosis to death or last follow-up and was compared using Kaplan–Meier method with the log-rank test for assessment of statistical significance.

Associations of TROP2 expression in primary tumor samples with tumor size, distant and lymph node metastasis, lymphonodal status, vascular and lymphatic infiltration, TROP2 expression in lymphonodal samples, Lauren classification, grading, combined positivity score of PD-L1 expression on tumor and immune cells (CPS), Her2-neu positivity, Claudin 18.2 positivity and mismatch-repair-status (MSI) were tested using the *χ*^2^ test. If the expected frequency in a cell was less than 5, Fisher's exact test was used. Effect size of nominal correlations was evaluated using Cramer’s V. Correlations of categorical variables were tested by Spearman correlation.

## Results

### TROP2 expression in primary tumors samples and correlation with survival

Of 314 primary tumor samples, 250 (79,6%) were evaluable after staining with Anti-TROP2. Low expression was observed in 145 samples (58%), moderate expression in 55 samples (22%) and high expression in 50 samples (20%). Thirty-five patients showed no TROP2 expression (14%), which resulted in an overall TROP2 positivity of 86%.

**Fig. 2:**
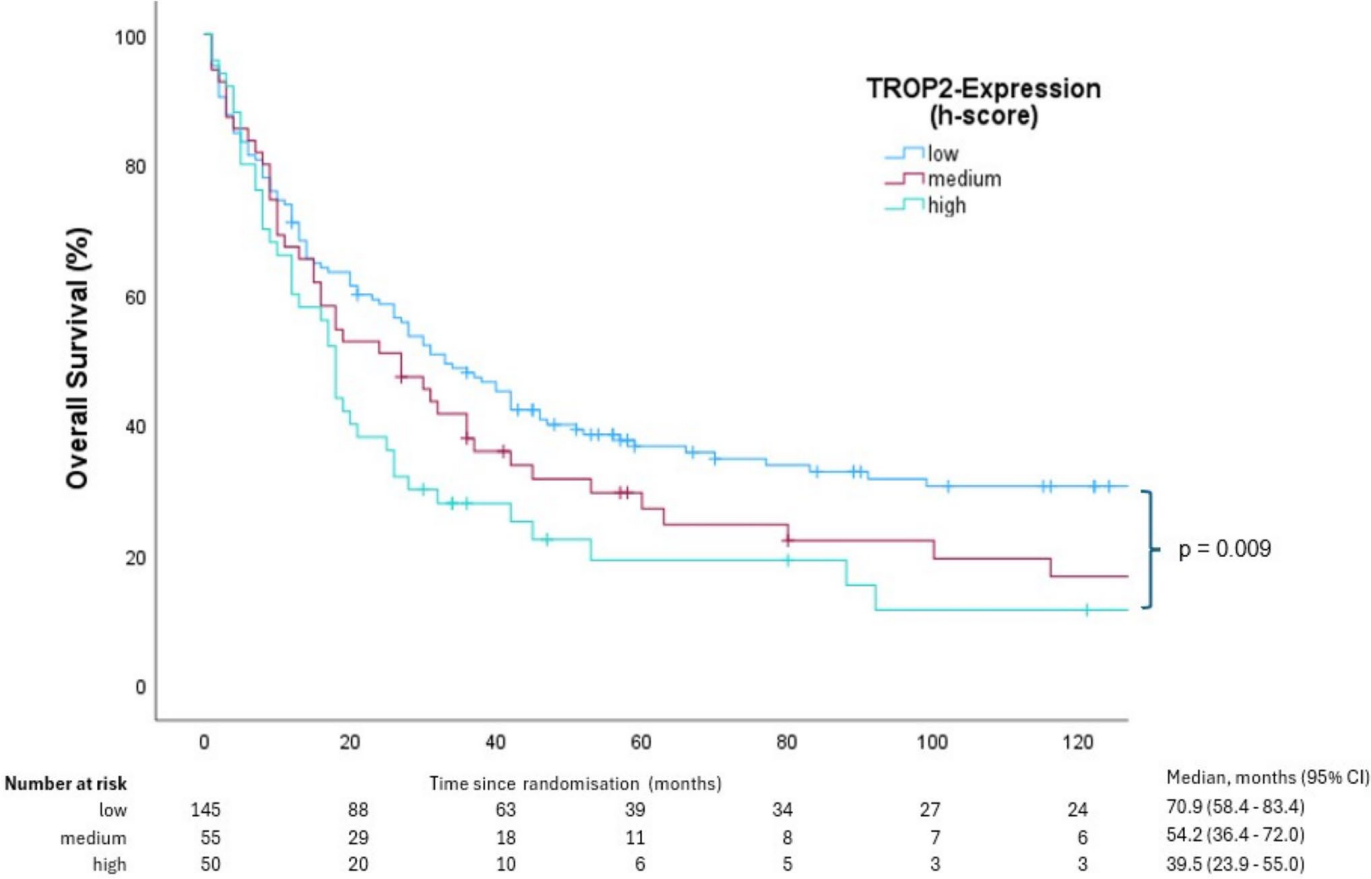
Kaplan–Meier plots of overall survival, divided by intensity of TROP2 expression. A vertical line marks a censored patient. There is a significant (log-rank X^2^ = 6.682, df = 1, p = 0.009) difference in survival between the low- and high-TROP2-expressing patients. There was no significant difference between the low- and medium-TROP2-expressing patients and the medium- and high-TROP2-expressing patients, respectively

There was a significant difference in survival between the low- and high-TROP2-expressing patients (70.9 months (95% KI 58.4–83.4) versus 39.5 months (95% KI 23.9–55.0) *p* = 0.009) (Fig. 2).

### TROP2 expression and clinicopathological features

Patients were stratified by H-score into three subgroups and were correlated with clinicopathological features (Table 1). Patients with high TROP2 expression showed statistically significant more tumor-related deaths (*p* = 0.013, *V* = 0.189), a worse tumor grading (*p* = 0.037, *V* = 0.143), and higher rates of lymphatic invasion (*p* = 0.05, *V* = 0.161). In addition, there was a non-significant trend towards increased positive nodal status (*p* = 0.093, *V* = 0.138) in patients with higher TROP2 expression. There were no significant differences in TROP2 expression and sex, age, TNM stage, localization, Lauren classification, vascular invasion, CPS, Claudin 18.2 expression, Her2/neu expression, and MSI.

### TROP2 expression in lymph node metastasis

Of 151 lymph node metastasis specimens, TROP2 immunohistochemistry was assessable in 107 (70.9%). Overall, 81.3% of evaluable samples demonstrated TROP2 positivity. Low-, medium-, and high-TROP2 expression levels were observed in 55 (51.4%), 30 (28.0%), and 22 (20.6%) patients, respectively. Sixty-two lymph node specimens were matched with corresponding primary tumor samples from the same individuals. The intensity of TROP2 expression showed a strong correlation between primary tumors and their corresponding lymph node metastasis (Table 2; Spearman’s ρ = 0.495; *p* < 0.001).

**Table 2:** TROP2 expression in primary tumor samples (*T*) and corresponding lymph node metastasis (*N*)

		TROP2 expression (*N*)					
		Low	Medium	High	N total	*p* value	Spearman
TROP2 expression (*T*)	Low	24	4	4	32 (51.6%)	< 0.001	correlation
	Medium	7	8	1	16 (25.8%)		0.495
	High	2	4	8	14 (22.6%)		
		33 (53.2%)	16 (25.8%)	13 (21%)	62 (100%)		

*H*-score groups between tumor samples and lymph node samples are correlated

## Discussion

### TROP2 is strongly expressed in AEG/S and correlates with worse outcome

To our knowledge, this study represents the first large Caucasian AEG/S cohort comprehensively profiled for TROP2 expression and correlated with detailed clinicopathological parameters. TROP2 was expressed in majority of primary tumors as well as in lymph node metastases. Higher TROP2 expression was significantly associated with increased lymphatic invasion, poorer tumor differentiation, a greater number of tumor-related deaths, and reduced overall survival. Although lymph node positivity was more frequent in cases with elevated TROP2 expression, this difference did not reach statistical significance.

The underlying pathobiology of worse outcomes could be explained by gene expression profiles in TROP2-positive cells that contribute to epithelial–mesenchymal transition, migration/invasiveness, and extracellular matrix interaction/remodeling. This mechanism has been observed in colorectal cancer, where TROP2 overexpression leads to lymph node metastases and poor tumor differentiation [20]. Supporting this hypothesis, we observed high TROP2 expression rates in the lymph node metastases that correlated with the intensity of expression in corresponding primary tumor samples.

### Differences of TROP2 expression in Asian and Caucasian cohorts

The present findings are broadly concordant with those reported by Zhao and colleagues [15], who analyzed a Chinese cohort of 600 gastric cancer patients and demonstrated that high TROP2 expression was associated with significantly poorer outcomes, including increased rates of lymph node or distant metastasis and a higher prevalence of intestinal-type tumors. These discrepancies between cohorts may be influenced by differences in the molecular profile of AEG/S in Asian and Caucasian patients [21] and, at least in part, by differences in *Helicobacter pylori* exposure. In the cohort studied by Zhao et al., 79.2% of patients were infected with *H. pylori*, whereas in Western populations, the prevalence is substantially lower, with only approximately 20% of non-cardia AEG/S cases attributable to *H. pylori* infection [20]. Mechanistically, TROP2—typically absent or minimally expressed in normal gastric mucosa—is upregulated during the metaplasia–dysplasia transition, a process frequently initiated and sustained by chronic *H. pylori*-induced inflammation [21]. Unfortunately, *H. pylori* infection status was not available for our cohort, precluding direct assessment of its role in modulating TROP2 expression. Moreover, Zhao et al. applied a dichotomous classification of TROP2 expression (high: H-score > 130; low: *H*-score < 130 or negative), whereas contemporary practice typically employs a three-tiered H-score system (low, medium, high) [18, 19].

Kim et al. [22] were able to show similar findings in their Korean cohort that comprised 412 surgically treated gastric cancer patients. They also found significantly poorer outcomes, higher rates of lymph node or distant metastasis and a higher prevalence of intestinal-type tumors in high TROP2-expressing patients, using a dichotomous H-score system.

### TROP2 expression as a potential biomarker

Kim et al. [22] reported significant associations between TROP2 expression and the predictive biomarkers HER2/neu and PD-L1. In our cohort, despite comparable frequencies of PD-L1–positive and HER2/neu-positive tumors, we were unable to replicate these findings. When applying a dichotomous H-score classification, we observed a trend toward higher PD-L1 expression in TROP2-high tumors (*p* = 0.073; data not shown), which may partially account for this discrepancy. Claudin 18.2, another emerging predictive biomarker, has not been shown to correlate with other biomarkers [5, 22], which appears to hold true for TROP2 in our dataset as well. Similarly, MSI status demonstrated no significant association with TROP2 expression, consistent with the observations of Kim et al. [22].

Compared with established predictive biomarkers such as HER2/neu and PD-L1, TROP2 appears to function primarily as a prognostic marker, given its association with reduced survival in patients exhibiting higher expression levels. Its potential role as a predictive biomarker is currently under investigation in ongoing clinical trials, including studies of anti-TROP2 antibody–drug conjugates in AEG/S, such as the SAGA trial (AIO-STO-0123/ass).

## Conclusion

TROP2 is highly expressed in AEG/S and is associated with increased lymphatic invasion, poor tumor differentiation, and reduced survival, supporting its role as a prognostic biomarker. Expression patterns in Caucasian cohorts align with findings from Asian populations, though differences in *H. pylori* exposure may contribute to variability. TROP2 showed no consistent correlation with HER2/neu, PD-L1, Claudin 18.2, or MSI, but ongoing trials, including the SAGA study (AIO-STO-0123/ass), will clarify its potential as a predictive biomarker and therapeutic target.

## Data Availability

The data that support the findings of this study are available from the corresponding author, AP, upon reasonable request.
